# Site‐Specific Surface Modification via Chemoselective Click Reactions on Cyclobutene‐Functionalized Monolayers

**DOI:** 10.1002/open.202500617

**Published:** 2026-03-30

**Authors:** Boone W. Evans, Kenneth N. Hipp, William D. Lambert, Wantanee Sittiwong, Rebecca Y. Lai, Patrick H. Dussault

**Affiliations:** ^1^ Department of Chemistry University of Nebraska–Lincoln Lincoln Nebraska USA

**Keywords:** click, cyclobutene, electrochemical sensing, inverse electron‐demand Diels–Alder, self‐assembled monolayers

## Abstract

ABSTRACT We report the first example of self‐assembled monolayers (SAMs) incorporating a cyclobutene‐substituted alkanethiol on a gold surface and the efficient modification of these monolayers via inverse electron‐demand Diels–Alder (IEDDA) cycloadditions. Mixed SAMs incorporating both cyclobutene‐ and azide‐substituted alkane thiols undergo controlled bisfunctionalization through either sequential or simultaneous performance of IEDDA and copper‐assisted alkyne/azide cycloaddition (CuAAC) ligations.

**Abbreviations**
ACV: alternating current voltammetry
C6‐OH: mercaptohexanol
C8‐CB: cyclobutenyl octanethiol
C8‐N3: 8‐azido octanethiol
CAN: ceric ammonium nitrate
CuAAC: copper‐promoted alkyne‐azide cycloaddition
DMF: *N*,*N‐*dimethylformamide
DMSO: dimethyl sulfoxide
EA: ethyl acetate
Hex: hexane
IEDDA: inverse electron‐demand Diels‐Alder cycloaddition
rt: room temperature
TBTA: tris(benzyltriazolylmethyl)amine

## 1 Introduction

Self‐assembled monolayers (SAMs) generated by the absorption of thiol‐containing amphiphiles and analytes onto a gold surface are an important platform for the development of biosensors, including electrochemical biosensors capable of detection of a wide range of analytes [1, 2]. We have been particularly interested in sensors based upon functionalized monolayers in which binding or release of an analyte controls distance of an electroactive component from the Au surface [3, 4]. Direct generation of functionalized thiol/gold SAMs requires the availability of precursors tethering a mercaptoalkyl group to the desired functionality. We and others have demonstrated the utility of an indirect approach based upon use of “click” chemistry to attach sensor components to the surface of SAMs bearing terminal azides or other functionality [5, 6, 7]. In this work, we explore the hypothesis that orthogonal click reactions can be employed sequentially or in parallel to selectively address the surface elements of a bisfunctionalized SAM [8].

Copper‐promoted alkyne‐azide cycloaddition (CuAAC), a rapid and highly specific transformation, has been the most frequently applied click reaction for functionalization of monolayers [9, 10]. However, there is growing interest in application of the inverse electron‐demand Diels‐Alder cycloaddition (IEDDA) between alkenes and heteroarenes [11, 12]. A particular advantage of IEDDA‐based functionalization is the ability to achieve high specificity and rapid reaction in the absence of a catalyst. We were particularly interested in the use of cyclobutenes as reactive partners for IEDDA reactions on surfaces given their modest cross‐section, reasonable chemical and thermal stability, and high reactivity toward 1,2,4,5‐tetrazines [11, 12, 13, 14, 15]. We were particularly interested in determining whether CuAAC and IEDDA click processes could be paired to achieve orthogonal ligation on a polyfunctionalized SAM (for examples of selective click reactions on SAMs, see: [16, 17]). We now describe the synthesis of a cyclobutenyl‐terminated mercaptoalkane, the unprecedented application of this substrate to preparation of cyclobutene‐functionalized Au/thiol SAMs, and the successful performance of sequential or simultaneous IEDDA and CuAAC functionalization of SAMs displaying both cyclobutene and azide groups at the solvent interface.

## 2 Materials and Methods

General synthetic conditions and procedures for preparation of electrode surfaces are described in Supporting Information.

### 2.1 Monolayer Formation

SAM formation was carried out through immersive deposition. The cleaned and characterized electrode was incubated at 4°C for the indicated period with 100 µL of a 10 mM PBS 1 M NaClO_4_ buffer containing one of two combinations of thiols. Single “click” monolayer systems were generated by incubating the electrodes overnight at 4°C with buffer containing 1 mM 6‐mercapto‐1‐hexanol (C6‐OH) as a passivating diluent and either 0.5 mM 8‐mercaptooctyl 3‐cyclobutene (C8‐CB) or 1 mM 8‐mercaptooctyl‐1‐azide (C8‐N_3_) as reactive components for the IEDDA or CuAAC click reactions, respectively. Dual “click” monolayer systems were generated analogously upon simultaneous exposure to buffer containing 1 mM C6‐OH, 0.5 mM C8‐CB, and 0.5 mM C8‐N_3_.

### 2.2 Click Reactions

*CuAAC* (*Azide/Alkyne*) cycloadditions were performed in the presence of varying concentrations of the alkyne component in 1:1 DMSO/H_2_O (DI) in the presence of 2 mM CuSO_4_ as the precatalyst, 0.5 mM sodium ascorbate (Na Ascorbate) as the reducing agent, and 2 mM tris(benzyltriazolylmethyl)amine (TBTA) as a reaction promoter which solubilizes and stabilizes Cu(I) in aqueous environments. The default concentration of the alkyne component was 25 µM (**Alk‐MB)** or 2 mM (**Alk‐FC)**; alterations of these concentrations are discussed within specific sections.

*Cyclobutene/Tetrazine* (IEDDA) cycloadditions were conducted in 40% CH_3_CN/H_2_O (DI) with either 0.2 µM **TZ‐Fc** or 1 µM **Tet‐MB**.

*Parallel CuAAC and IEDDA functionalization* was conducted in 44/44/12 or 47/47/6 DMSO/H_2_O/ACN (tetrazine is dissolved in ACN) containing 2 mM CuSO_4_, 0.5 mM Na ascorbate, and 2 mM TBTA. The solution concentrations of the reaction partners (alkyne for CuAAC, tetrazine for IEDDA) were optimized as described in Results and Discussion.

Reactions were conducted for 30 min unless indicated and then the electrodes were rinsed as described above.

### 2.3 Electrochemical Characterization

Electrochemical measurements were carried out with a standard three‐electrode electrochemical cell comprising six polycrystalline gold disk working electrodes with a geometric area of 0.0314 cm^2^, a platinum wire counter electrode, and an Ag/AgCl reference electrode. All electrodes used in characterization were purchased from CH Instruments (Austin, TX). Measurements were performed using an eight‐channel CHI 1040A electrochemical workstation (CH Instruments, Austin, TX). Alternating current voltammetry (ACV) and cyclic voltammetry (CV) were used for system characterization. The electrolyte solution used for characterization of the system was a 10 mM PBS 1 M NaClO_4_ buffer at pH 7.3. NaClO_4_ was used in place of other salt options due to the susceptibility of ferrocenium toward attack by nucleophilic ions [18, 19].

### 2.4 Capacitance

Capacitance values, used to determine the packing of monolayers, was determined by the following equation: Absolute(anodic + cathodic current)/(2*scan rate*geometric area). CV was taken from 0 to 200 mV at 51.2 V/s. The anodic and cathodic current recorded was taken at 50 mV. A lower capacitance value is indicative of a well‐packed monolayer.

### 2.5 Coverage/Probe Density

Coverage was determined through CV characterization [20]. The charge under the redox label reduction peak in CVs was collected at slow scans rates (20, 50, 100 mV/s) and applied to the following equation: Γ = *Q*/*nFA*, where Γ is the probe density, *Q* is the charge of the reduction peak, *n* is the number of electrons transferred (2 or MB, 1 for Fc), *F* is the Faraday's constant, and *A* is the calculated real area of the electrode.

### 2.6 Behavior

ACVs were taken to characterize the stability and behavior of the finalized “click” platform. The MB peak was seen at approximately −0.25 V and the Fc peak was present at approximately 0.33 V. The peak current was used to record the signature of the redox label. Representative ACVs were taken at 70 Hz as this frequency resulted in a similar peak current for the two redox indicators. However, as frequency increased, the size of the Fc peak increased at a faster rate than the MB peak due to the faster single‐electron transfer.

## 3 Results and Discussion

**FIGURE 1:**
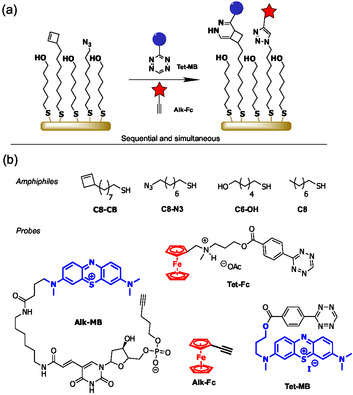
(a) Overview of surface functionalization reactions; (b) structures of reaction partners.

Our investigations, overviewed in Figure 1, employed functionalized SAMs derived from deposition of mercaptohexanol (**C6‐OH**) plus cyclobutenyl octanethiol (**C8‐CB**) and/or 8‐azido octanethiol (**C8‐N3**) onto a polished Au surface. Although the use of longer alkyl backbones is associated with increased SAM stability, previous work on SAM‐based electrochemical sensors has demonstrated that use of linkers containing ten or eleven CH_2_ units, linkers results in greatly reduced current [21, 22]. The ease and efficiency of IEDDA and/or CuAAC functionalization was probed using a common set of electrochemical reporters based upon tetrazine or alkyne conjugates of ferrocene (**Tet‐Fc, Alk‐FC**) and the azure B skeleton (**Tet‐MB, Alk‐MB**). IEDDA surface reactions were initially investigated on SAMs containing **C8‐CB**; sequential and parallel IEDDA/CuAAC reactions were subsequentially investigated on SAMs containing both **C8‐CB** and **C8‐N3**.

Synthesis of reaction components is overviewed here and described in detail within Supporting Information. **C8‐N3** was prepared based upon modifications of reported procedures [23]. **C8‐CB** was prepared by an extension of a reported sequence [24]. Although it contains both a thiol and a reactive alkene, **C8‐CB** proved stable to isolation, purification, and storage at −20°C. The alkyne‐containing probes **Alk‐MB** and **Alk‐Fc** are commercially available. Synthesis of the tetrazine‐containing probes proved unexpectedly challenging. Attempted synthesis of **Tet‐MB** via esterification of a *N*‐2‐hydroxyethyl derivative of methylene blue with 4‐tetrazinyl benzoic acid failed under some common approaches (dicyclohexyl carbodiimide, preformation of acid chloride, Mitsunobu activation) but was accomplished via base‐promoted reaction of the alcohol with the mixed anhydride derived from activation of the 4‐tetrazinyl benzoic acid with trichlorobenzoyl chloride [25, 26]. The introduction of the ester linkage of **Tet‐Fc** was unsuccessful using Mitsunobu activation or a preformed acid chloride. Reaction was possible using dicyclohexyl carbodiimide, but the products were contaminated with impurities that were difficult to remove. Esterification was ultimately accomplished in modest yield through coupling the preformed hydroxy succinimide (NHS) ester of 4‐tetrazinyl benzoic acid with the alcohol‐containing sidechain of a functionalized ferrocene. The free base of **Tet‐Fc** was prone to decomposition, even when held at low temperatures under an inert atmosphere. Fortunately, the readily prepared acetate salt was far more stable and also offered superior water solubility [27].

### 3.1 Initial Screening of Conditions

**FIGURE 2:**
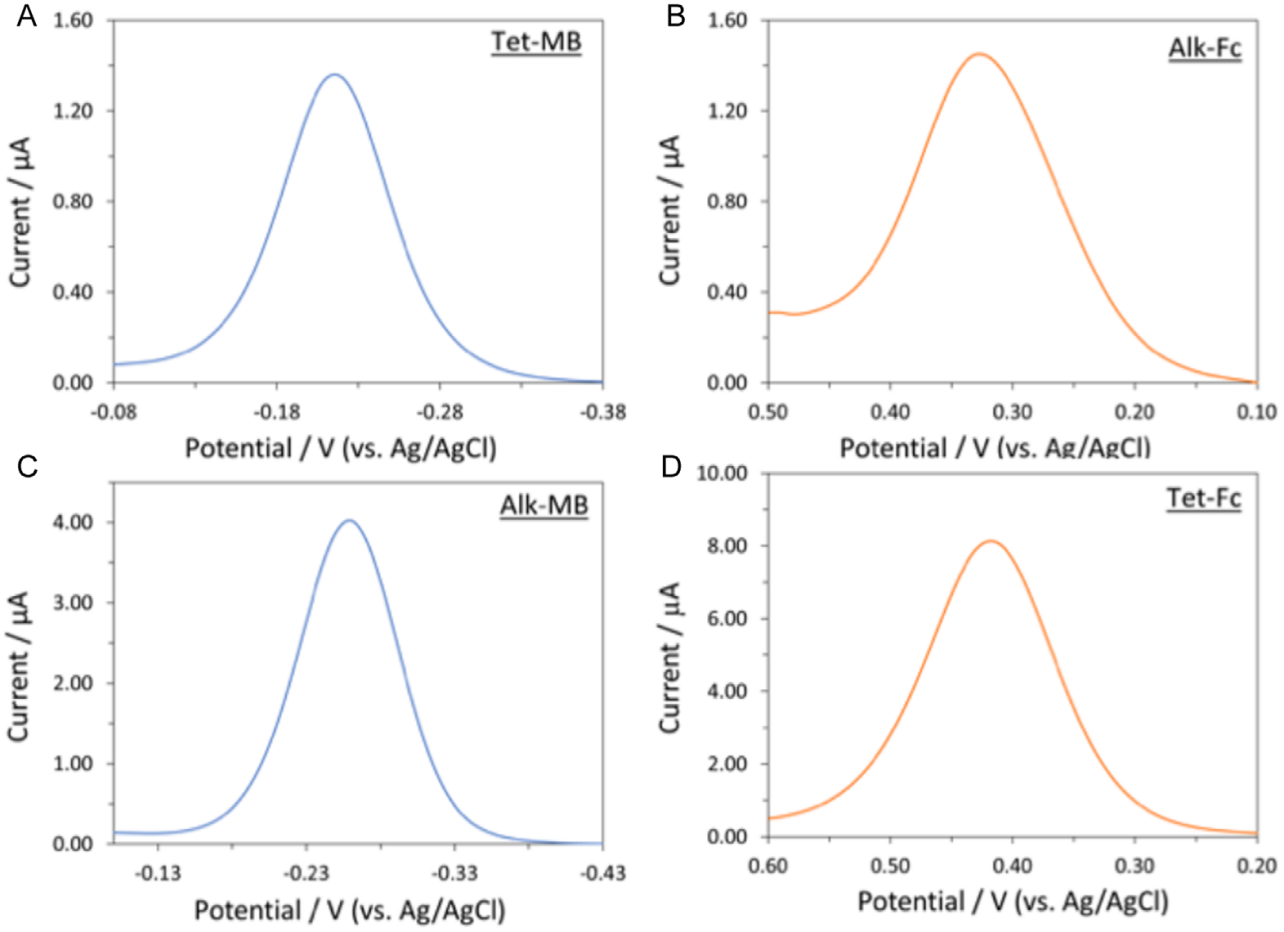
ACVs recorded at 70 Hz in PBS‐7.3 for (A) a **C8‐CB/C6‐OH** monolayer with **Tet‐MB**, (B) a **C8‐N3/C6‐OH** monolayer with **Alk‐Fc**, (C) a **C8‐N3/C6‐OH** monolayer with **Alk‐MB**, and (D) a **C8‐CB/C6‐OH** monolayer with **Tet‐Fc.** MB‐related ACVs shown in blue; FC‐related ACVs in red.

Our initial screening of conditions for IEDDA reaction on a SAM incorporating **C8‐CB** demonstrated that a universal postincubation rinse sequence was effective in removing both hydrophilic and hydrophobic adsorbates, enabling differentiation of covalent ligation and nonspecific absorption (see SI for details) [6, 28]. With an effective reaction and workup protocol in place, individual IEDDA and CuAAC click reactions were tested on the complementary monofunctional monolayer electrodes, **C8‐CB/C6‐OH** and **C8‐N3/C6‐OH**, respectively. The presence of the redox peaks corresponding to MB at ∼−0.23 V and Fc at ∼0.35 V in the ACV spectra indicated that in each case we achieved successful conjugation of the redox labels to the functionalized surfaces (Figure 2).

### 3.2 Sequential “Click” of Bifunctional Monolayers

**FIGURE 3:**
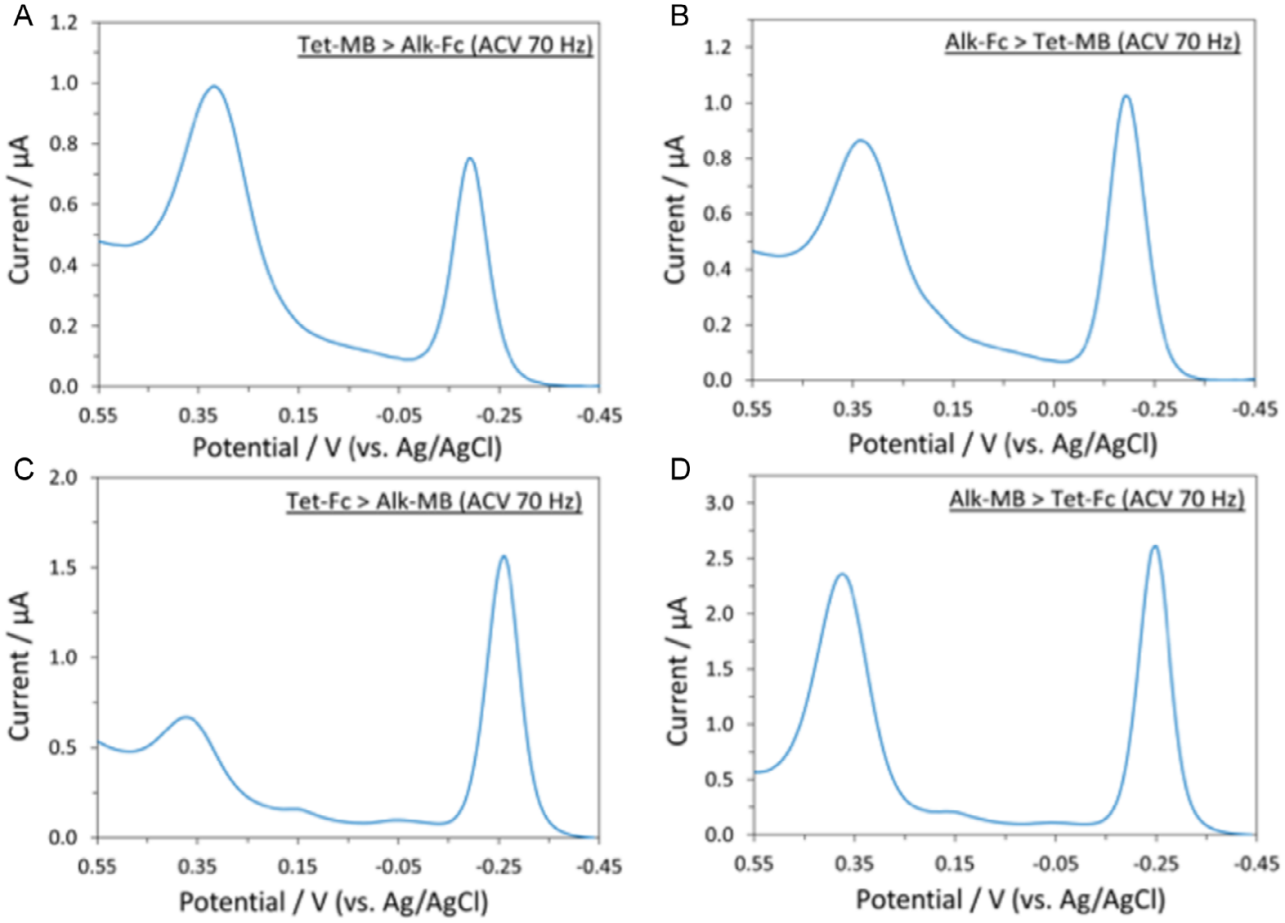
ACVs of a mixed C8‐CB/C8‐N3 SAM after sequential addition of the two redox labels via CuAAC and IEDDA “click” reactions. (A) **Tet‐MB** followed by **Alk‐Fc**. (B) **Alk‐Fc** followed by **Tet‐MB**. (C) **Tet‐Fc** followed by **Alk‐MB**. (D) **Alk‐MB** followed by **Tet‐Fc**.

After successfully demonstrating IEDDA and CuAAC reactions on monofunctionalized monolayers containing either **C8‐CB** and **C8‐N3**, we explored the efficiency of sequential IEDDA and CuAAC click reactions on a monolayer containing both clickable amphiphiles. Four sets of conditions were attempted: (A) Addition of **Tet‐MB** followed by **Alk‐Fc**; (B) Addition of **Alk‐Fc** followed by **Tet‐MB**; (C) Addition of **Tet‐Fc** followed by **Alk‐MB;** (D) Addition of **Alk‐MB** followed by **Tet‐Fc**. The AVC spectra (Figure 3) indicated all four variants of the sequential click sequences were successful, demonstrating that the nature of the first‐introduced redox label had limited impact on the efficiency of the second coupling. Each redox label was determined to be present at 1.0–3.0 × 10^12^ molecules/cm^2^, a coverage range relevant to many surface‐immobilized biosensors [29, 30, 31, 32, 33]. This result shows the potential of a bifunctional SAM for creating sensors capable of sequential detection of two different targets based upon orthogonal click reactions.

### 3.3 Parallel (Simultaneous) Click Reactions of Bis Functionalized Monolayers

**FIGURE 4:**
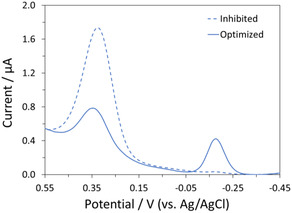
Optimization of parallel click IEEDA/CuAAC reaction showcasing the comparison between the initial attempt and the optimized conditions.

We next explored performance of CuAAC and IEDDA “click” reactions in parallel. Our initial approach incubated the bifunctional SAM with a reactant solution containing **Alk‐Fc**, **Tet‐MB**, Cu(II), ascorbate, and TBTA. Following a rinse to remove noncovalently attached materials, we observed a substantial signal corresponding to the redox cycle of Fc and the absence of a corresponding signal for MB (Figure 4); the results suggested inhibition of the IEDDA ligation by one of the reagents or intermediates associated with the CuAAC click. To pinpoint the source of the inhibition, the IEDDA functionalization of **C6‐OH**/**C8‐CB** with **Tet‐MB** was investigated in the presence of individual reagents associated with the CuAAC reaction (see the SI section for details). Significant inhibition was observed in the presence of added Cu(I) or the combination of Cu(II) and ascorbate; both observations point strongly to inhibition by Cu(I).

The apparent inhibition of the IEDDA reactions by Cu(I) is intriguing. Complexation of alkenes by copper ions is well known and, it is tempting to attribute the lowered IEDDA reactivity to stabilization provided by a metal complex [34]. However, Cu(I), along with Lewis acidic metal ions such as Au(I) and Ag(I), has been observed to catalyze IEDDA reactions of heterodienes, presumably by lowering the energy of the lowest unoccupied molecular orbital (LUMO) [35]. Moreover, IEDDA and CuAAC reactions have been performed in parallel in solution [8, 36]. We note that deactivation of tetrazines through reduction has been reported in the presence of a copper source and ascorbate [37].

**FIGURE 5:**
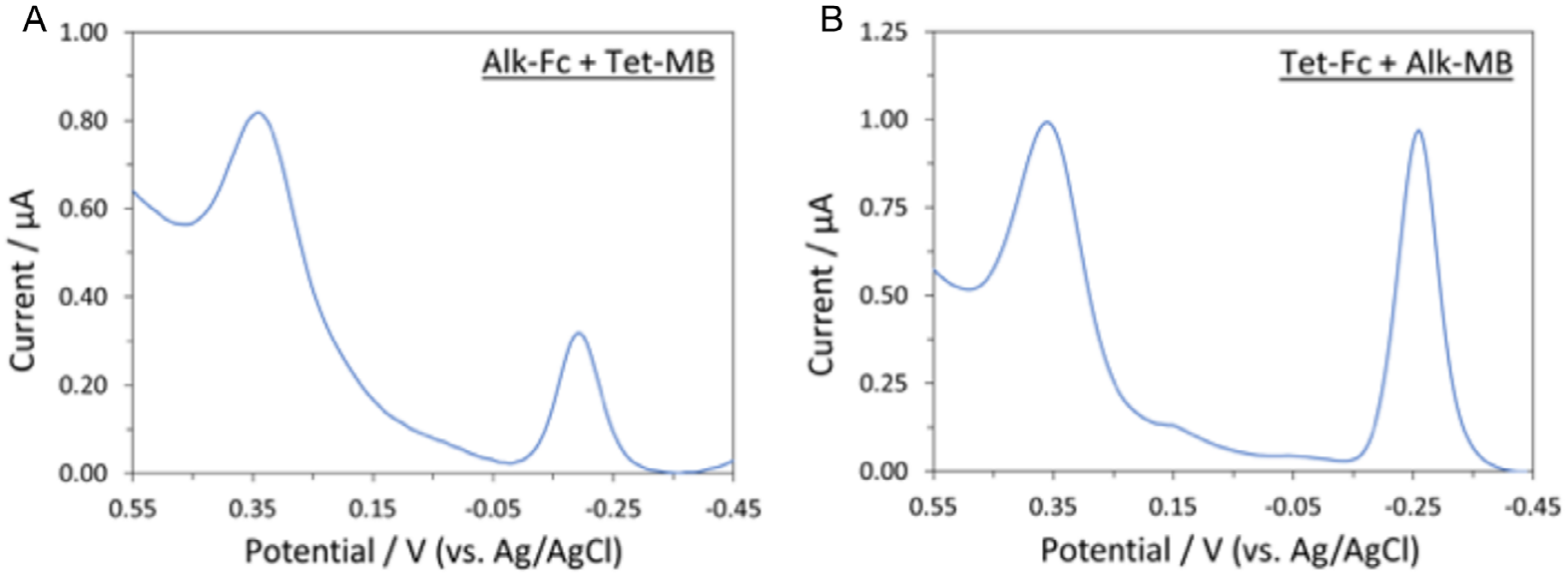
Representative ACVs for parallel conjugation: (A) **Alk‐Fc** and **Tet‐MB** and (B) **Tet‐Fc** and **Alk‐MB** following optimization. Scans taken at 70 Hz.

With a goal of identifying conditions to achieve coverage ≥ 1.0 × 10^12^ molecules/cm^2^ for both the IEDDA and CuAAC conjugations, we explored the influence of reagent concentration and stoichiometry on the efficiencies of the conjugations involving **Alk‐Fc**/**Tet‐MB** and **Tet‐Fc**/**Alk‐MB** (Figure 5). Both sets of reactions employed 2 mM CuSO_4_, 0.5 mM Na ascorbate, and 2 mM TBTA. The concentration of the redox probes proved critical to successful reaction. For the combination **Tet‐MB**/**Alk‐Fc**, ideal behavior was attained with 50 µM of each component. For **Tet‐Fc/Alk‐MB**, ideal behavior was attained with 12 and 25 µM concentrations, respectively. The results demonstrate that a simple optimization of the concentrations of the tetrazine containing components (**Tet‐MB** and **Tet‐Fc**) is able to overcome inhibition and provide the desired surface coverage.

## 4 Conclusion

IEDDA and CuAAC click reactions can be performed sequentially or in parallel on SAMs displaying both azide and cyclobutene components to achieve surface coverages suitable for sensing applications. Although the CuAAC and IEDDA processes are not completely orthogonal, the partial inhibition of IEDDA reactions by Cu(I) during parallel functionalization can be overcome by appropriate choice of conditions. Our discovery suggests new possibilities for controlled generation of multifunctional surface‐based sensors.

## Supporting Information

Additional supporting information can be found online in the Supporting Information section. **Supporting Figure SI‐1**: (A) Representative ACV showing the nonspecific adsorption of the **Tet‐MB** to a bare gold electrode compared to an electrode with the appropriate ‘click’ molecule. (B) Showcasing the effectiveness of various treatments to remove nonspecific adsorption. **Supporting Figure SI‐2**: Synthesis of **C8‐CB**. **Supporting Figure SI‐3**: Preparation of **C8‐N3**. **Supporting Figure SI‐4**: Preparation of **Tet‐MB**. **Supporting Figure SI‐5.** Preparation of **Tet‐Fc**. **Supporting Table SI‐1.** Influence of additives on IEDDA click.

## Author Contributions

R.Y.L. and P.H.D. designed and oversaw the project. B.W.E., K.N.H., W.D.L., and W.S. conducted experimental work. All authors contributed to the interpretation of results. B.W.E. and K.N.H. wrote up portions of the work. R.Y.L., P.H.D., and W.S. took the lead in writing the manuscript.

## Funding

This research was supported by the National Science Foundation (EPS‐1004094) and the University of Nebraska‐Lincoln. A helium recovery system supporting the Bruker NMR spectrometers was purchased with support from the NCIBC Systems Biology Core (NIH NIGMS P20 GM113126). Portions of this work were conducted in facilities that were remodeled support from the National Institutes of Health (RR016544). WDL received support through the Ronald E. McNair Scholars program.

## Conflicts of Interest

The authors declare no conflicts of interest.

## Supporting information

Supplementary Material

## Data Availability

The data that support the findings of this study are available in the supplementary material of this article.
