# Supplementary material for: Site‐Specific Surface Modification via Chemoselective Click Reactions on Cyclobutene‐Functionalized Monolayers
Source: ChemistryOpen. 2026 Mar 30;15(4):e202500617. doi: 10.1002/open.202500617 (PMC13140894; doi:10.1002/open.202500617)
Supplement: Supplementary file 1 — Supplementary Material [file OPEN-15-e202500617-s001.pdf]

SUPPORTING INFORMATION

Site-specific surface modification via chemoselective click reactions on cyclobutene-functionalized monolayers.

Evans, B.; Hipp, K. N.; Lambert, W. D.; Sittiwong, W.; Lai, R. Y.; Dussault, P. H.

pdussault1@unl.edu

GENERAL SYNTHETIC PROCEDURES 2
SURFACE PREPARATION 2
INVESTIGATION OF CONDITIONS FOR CLICK REACTIONS 2
  Optimization of procedures for removal of nonspecifically absorbed materials 2
  Determination of reactant compatibility during parallel reactions 3
SYNTHETIC PROCEDURES 4
  Synthesis of mercaptoalkyl cyclobutene (**C8-CB**) 4
  Synthesis of 8-azidooctanethiol (**C8-N3**) 5
  Synthesis of **Tet-MB** 7
  Synthesis of **Tet-Fc** 10

GENERAL SYNTHETIC PROCEDURES

Reagents and solvents were used as supplied commercially, with the exception of THF (distilled from Na/Ph_2CO); CH_2Cl_2 (distilled from CaH_2) and DMF (purchased as anhydrous or distilled at reduced pressure from CaH_2; stored over activated 4 Å molecular sieves). Reagents supplied as solutions were dispensed based upon manufacturer-supplied concentrations. Prepared solutions employed the indicated solvent and/or water (DI, milliohm). Synthetic reactions were conducted at room temperature under an atmosphere of N_2 in a round-bottom flask equipped with a magnetic stir bar except as noted. Thin layer chromatography (TLC) was performed on 0.25 mm hard-layer silica G plates with developed plates visualized under a UV lamp or by staining: 1% ceric sulfate and 10% ammonium molybdate in 10% H_2SO_4 (general stain, after heating); 3% vanillin in 3% H_2SO_4 in EtOH (general stain, after heating); aq. $KMnO_4$ (alkenes). Chromatography was conducted on 40-63 mm silica . Unless otherwise noted, NMR spectra were acquired in $CDCl_3$, with ^1H spectra reported by chemical shift (multiplicity, J couplings in Hz, number of protons). IR spectra were recorded as neat films on a ZrSe crystal; selected absorbances are reported in cm^{-1}. Details related to substrate synthesis and characterization are provided in Supporting Information.

SURFACE PREPARATION

Electrode surfaces were prepared using a previously described method.[1] The electrode surface was polished using a diamond slurry (Buehler), rinsed and sonicated in DI water to remove residual diamond particulates, and then electrochemically cleaned through oxidation and reduction cycles in 0.5 M H_2SO_4. The electrochemically active area of the surface was determined by comparison of the charge density associated with the gold oxide reduction peak obtained in a 0.05 M H_2SO_4 solution relative to the reported value of 400 µC cm^{-2}. Using the obtained value, the roughness factor (ρ = real area / geometric area) was determined; electrodes with rho factors of ~ 1.05 ± 0.15 were accepted for modification. After area determination the electrodes were cleaned with a 15 sec DI H_2O rinse and dried with nitrogen gas to await monolayer formation.

INVESTIGATION OF CONDITIONS FOR CLICK REACTIONS

Optimization of procedures for removal of nonspecifically absorbed materials

Preliminary attempts to explore conditions for surface IEDDA compared an electrode functionalized with **C8-CB** against one passivated with a mixture of **C8-Methyl** (1 mM) and **C6-OH** (2 mM), a nonclickable coating designed to mimic the hydrophobicity of the cyclobutene-containing system in a system. The two electrodes were individually exposed to the **Tet-MB** probe followed by one of three different post "click" rinse procedures:

Method 1: Directly spraying the following reagents onto the electrode surface for 15 seconds: DI water, ethanol, 5% aq. Tween 20, ethanol, DI water. This protocol previously employed by one of our labs as a universal rinse for removal of both hydrophilic and hydrophobic adsorbates.[1,2]

Method 2: A 4-minute incubation in 0.5 M H_2SO_4, with the assumption that acidic conditions may protonate potential adsorbates and reduce their affinity for the monolayer.

Method 3: A 4-minute incubation in 40:60 acetonitrile (ACN):H_2O, the same solvent mixture used for the “click” reaction, as it could solubilize and remove some of the non-specifically adsorbed TZ-MB.

As illustrated in Figure **SI-1**, Method 1, sequential rinsing with water > ethanol > 5% tween > ethanol > water, proved the most effective in removing noncovalently bound probe.

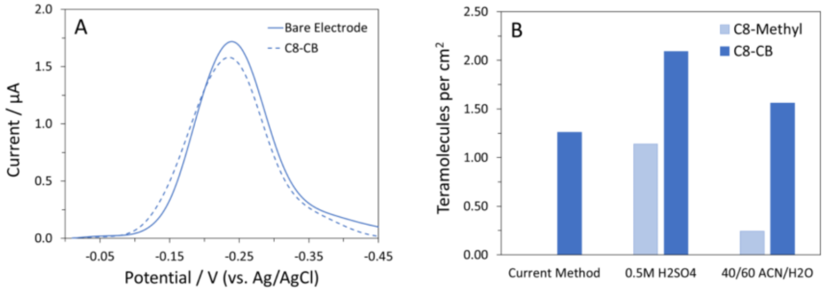

Figure SI-1: (A) Representative ACV showing the nonspecific adsorption of the **Tet-MB** to a bare gold electrode compared to an electrode with the appropriate “click” molecule. (B) Showcasing the effectiveness of various treatments to remove nonspecific adsorption.

Determination of reactant compatibility during parallel reactions

The determination on inhibition: The IEDDA functionalization of the **C8-CB** SAM was carried out under three sets of conditions, each based upon exposure of the SAM to dilute solutions of **Tet-MB** in 50/50 DMSO/H_2O containing three different sets of additives: 1) added Na ascorbate (solution 1), added $CuSO_4$ (solution 2), added Na ascorbate and $CuSO_4$ (solution 3). The presence of ascorbate (solution 1) had no detectable impact on the effectiveness of the IEDDA cycloaddition. The presence of $CuSO_4$ (solution 2) slightly reduced coverage from $1.5x10^{12}$ molecules/cm^2 to $1.0x10^{12}$ molecules/cm^2. $CuSO_4$ and the ascorbate reducing agent (solution 3) resulted in a substantially reduced coverage of $0.4x10^{12}$ molecules/cm^2. Based upon these results, we investigated the reaction in the presence of saturated aq. CuCl (solution 4) and observed coverage of $0.3x10^{12}$ molecules/cm^2. The results point strongly to Cu(I)-based inhibition of the IEDDA “click” reaction between **C8-CB** and **Tet-MB**.

Table SI-1. Influence of additives on IEDDA click.

Condition	DMSO/ H_2O	+ ascorbate	+ $CuSO_4$	+ ascorbate + $CuSO_4$	+ CuCl
Effectiveness on Click Reaction	No change	No change	Slightly inhibited	Inhibited	Inhibited
Coverage (molecules/cm^2)	$1.5x10^{12}$	$1.5x10^{12}$	$1.0x10^{12}$	$0.4x10^{12}$	$0.3x10^{12}$

SYNTHETIC PROCEDURES

Synthesis of mercaptoalkyl cyclobutene (**C8-CB**)

Synthesis of **C8-CB** was accomplished through a four-step sequence overviewed in Figure SI-2.

Figure SI-2. Synthesis of **C8-CB**

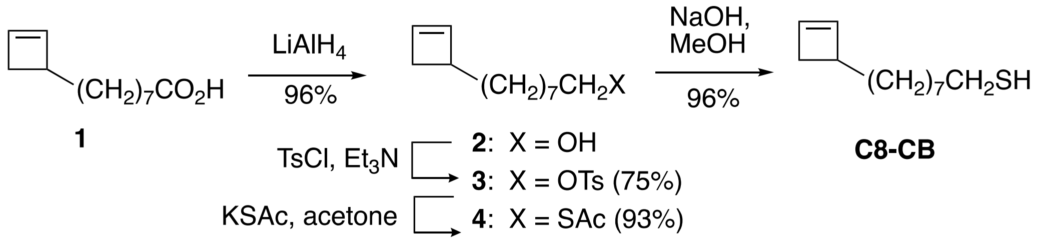

8-(Cyclobut-2-en-1-yl)octan-1-ol (**2**): $LiAlH_4$ (powder, 0.1632 g, 4.3 mmol, 5 eq.) was placed in a 3-neck round-bottom flask topped with a reflux condenser; the flask was evacuated and back-filled with N_2. The $LiAlH_4$ was suspended in 4 ml of ether and 0.86 mmol (0.1806 g, 1 eq.) of the cyclobutenyloctanoic acid (**1**) were added. The reaction was heated to a gentle reflux (40°C, oil bath) for 45 min, and then, after cooling, carefully quenched with water, resulting in a white suspension. Careful acidification to pH 4 with aqueous 1M HCl resulted in formation of a clear solution. The combined EA extracts (2 x 30ml) were washed with water (2 x 10ml), and the organic layer was dried with Na_2SO_4. Concentration *in vacuo* produced alcohol **2** as a clear oil (0.1615 g, 96%) that appeared pure by NMR and was used without further purification: R_f = 0.26 (10% EA/Hex) IR (thin film) 3348 (br), 2912 (s), 2851 (s) cm^{-1}; ^1H δ 1.29 (m, 10H), 1.44 (m, 2H), 1.56 (m, 2H), 2.04, (d, 1H, 13.4), 2.64 (dd, 1H, 13.3), 2.77 (m, 1H), 3.64 (t, 3H, 6.6) 6.04 (m, 1H) 6.10 (m, 1H); ^{13}C δ 25.9, 28.1, 29.5, 29.7, 29.8, 32.9, 34.8, 37.0, 44.4, 63.2, 135.2, 141.2. HRMS-EI calc. for $C_{12}H_{22}O$ $(M)^+$: 182.1671; found: 182.1671.

8-(Cyclobut-2-en-1-yl)octyl 4-methylbenzenesulfonate (**3**): To a solution of *p*-toluenesulfonyl chloride (0.2749 g, 1.5 mmol, 1.7 eq) in CH_2Cl_2 (0.5 ml) was added a solution of alcohol **2** (0.1615 g, 0.82 mmol, 1.0 equiv) in CH_2Cl_2 (1.25 ml), followed by Et_3N (0.20 mL, 1.4 mmol,1.75 eq; dropwise). Following the addition of the amine, the reaction was heated to 35°C (oil bath) for 4 h and then cooled to ~ 5°C (ice bath). The reaction was then quenched with sat. aq. $NaHCO_3$ (8 ml) and the mixture extracted with CH_2Cl_2 (3 x 20 ml). The combined organic layers, after being washed with water (1 x 10 ml), were dried with Na_2SO_4 and concentrated in vacuo. Purification by gradient silica flash column chromatography (1.8 x 15 cm column) using 1-3% ether/Hex generated, after concentration, 0.2163 g (75%) of **3** as a light yellow oil: R_f = 0.53 (10% EA/Hex); IR (thin film) 2920 (s), 2852 (m), 1359 (m), 1210, 1190 (s); ^1H δ 1.23 (m, 10H), 1.43 (m, 2H), 1.63 (p, 2H, 7.8), 2.04 (d, 1H, 13.4), 2.45 (s, 3H), 2.63 (dd, 1H, 13.3, 4.1), 2.76 (m, 1H), 4.01 (t, 2H, 6.54), 6.04 (d, 1H, 2.6) 6.10 (d, 1H, 2.6) , 7.34 (d, 2H, 8.1) and 7.79

(d, 2H, 8.3); ^{13}C δ 21.8, 25.4, 28.1, 28.9, 29.0, 29.5, 29.7, 34.7, 37.0, 44.3, 70.8, 128.0, 129.9, 133.4,135.3; HRMS ESI calc. for $C_{19}H_{28}NaO_3S$ $[M]^+$: 359.1657; found 359.1660.

***S*-(8-(Cyclobut-2-en-1-yl)octyl) ethanethioate (4):** A solution of potassium thioacetate (0.0883 g, 0.86 mmol,1.5 eq.) and sulfonate **3** (0.2008 g, 0.5737 mmol, 1.0 eq.) in acetone (2.5 mL) was heated to 30°C (oil bath) for 24 hours. Water (5 mL) was added, and the mixture was extracted with Hex (3 x 20 ml). The hexane extracts were washed with water (1 x 10 ml) and the combined extracts were dried with Na_2SO_4. The residue obtained after concentration in vacuo was purified by flash column chromatography (0.75 x 15 cm column) in 2% ether/Hex (~200 ml) to produce 3 (0.1335 g, 93%) as a light yellow oil: R_f = 0.74 (10% EA/Hex); IR (thin film) 2910 (s), 2851 (m), 1692 (m), 1177 (w), 697 (w); ^1H δ 1.28 (m, 8H), 1.34 (m, 2H) 1.44 (m, 2H), 1.56 (m, 2H), 2.04 (d, 13.4), 2.32 (s, 3H), 2.64 (dd, 1H, 4.1, 13.4) 2.76 (m, 1H), 2.86 (t, 2H, 7.4), 6.03 (d, 1H, 2.4) 6.10 (d, 1H, 2.5); ^{13}C δ 28.1, 28.9, 29.2, 29.3, 29.6, 29.6, 29.8, 30.8, 34.7, 37.0, 44.3, 135.2, 141.2, 196.2; HRMS-ESI calc. for $C_{14}H_{24}NaOS$ $[M]^+$: 263.1446; found: 263.1444.

8-(Cyclobut-2-en-1-yl)octane-1-thiol (C8-CB): A pellet of NaOH (0.1200 g, 3 mmol, 15 equiv) was added to a flame dried flask and dissolved in methanol (4 mL). Nitrogen was then bubbled through the solution for 10 minutes to remove oxygen. In a separate flask, a solution of thioacetate **3** (0.0479 g, 0.2 mmol, 1.0 equiv) in CH_2Cl_2 (3 mL) was sparged with a stream of nitrogen for 10 minutes. The methanolic base was added dropwise to the thioacetate solution by cannula and the reaction was stirred for 1 hour at rt. A solution of aq. HCl (6M, 3 mL, 18 mmol, 90 equiv), deoxygenated by sparging as described above, was then added to the reaction mixture by cannula. The reaction was diluted with DI H_2O (20 ml) and the mixture extracted with EA (3 x 10 ml). The organic layers were sequentially washed with DI H_2O (1 x 10ml) and brine (1 x 10ml), and the combined organic layer was dried with Na_2SO_4 and concentrated in vacuo. Purification by flash column chromatography (1.8 x 15 cm column) in Hex produced 0.0377 g (96%) of thiol **5** as a clear oil: R_f = 0.87 (10% EA/Hex); IR (thin film) 2910 (s), 2851 (m), 1692 (m), 1463 (w) cm^{-1}; ^1H δ 1.29 (m, 10H), 1.37 (m, 2H), 1.44 (m, 2H), 1.61 (m, 2H), 2.04 (d 1H, 13.4), 2.52 (q, 2H, 7.5), 2.64 (dd, 1H, 4.04, 13.3), 2.77 (m, 1H), 6.04 (d, 1H, 2.7) 6.10 (d, 1H, 2.6); ^{13}C δ 24.8, 28.1, 28.5, 29.2, 29.6, 29.8, 34.2, 34.8, 37.0, 44.4, 135.2, 141.2; HRMS-EI calc. for $C_{12}H_{22}S$ $(M)^+$: 198.1442; found: 198.1438.

Synthesis of 8-azidooctanethiol (**C8-N3**)

C8-N3, which has been reported without characterization, was prepared by the route shown in Figure SI-3.

Figure SI-3: Preparation of **C8-N3.**

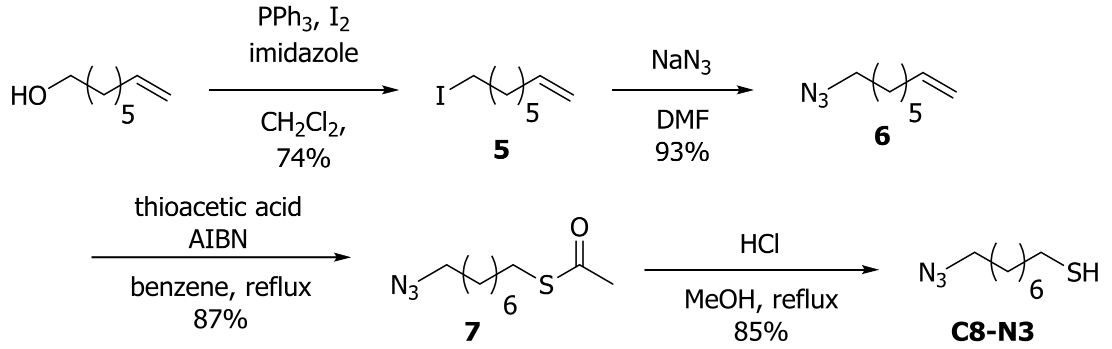

8-Iodooct-1-ene (5): A 100ml flask equipped with stir bar was flame dried under vacuum and back-filled with nitrogen. CH_2Cl_2 (25 ml), iodine (0.9518 g, 7.5 mmol, 1.5 eq.) triphenylphosphine (1.9672 g, 7.5 mmol, 1.5 eq.) and imidazole (0.5106 g, 7.5 mmol, 1.5 eq.) were sequentially added with stirring. After the mixture had stirred for 15 mi, 7-octen-1-ol (0.6411 g, 0.75 ml, 5 mmol, 1.0 eq.) was (dropwise, over minutes). TLC analysis showed the reaction to be incomplete after 18 h. Following a second addition of iodine (0.9518 g, 7.5 mmol, 1.5 eq.) triphenylphosphine (1.9672 g, 7.5 mmol, 1.5 eq.) imidazole (0.5106 g, 7.5 mmol, 1.5 eq.) and dichloromethane (6 ml), the reaction was stirred overnight at reflux. After a total of 40 h, no starting material was visible (TLC). The mixture was diluted with hexanes (40 ml) and the resulting suspension was cooled to 0°C and then vacuum filtered through a pad of Celite. The filtrate was concentrated in vacuo to produce a dark oil, which was purified by column chromatography (silica, 1.9 x 25 cm) using 2% MeOH in CH_2Cl_2 to afford, after concentration, 0.880 g (74%) of (**5**) as a clear oil. Spectral data appeared identical to those in previous reports.[3] R_f = 0.93 (10% EA:Hex); ^{1}H δ 1.30-1.44 (m, 6H), 1.82 (p, 2H, 7.1), 2.05 (q, 2H, 7.0), 3.19 (t, 2H, 7.0), 4.94, (bd, 1H, 10.2), 5.00 (bd, 1H, 17.1), 5.80 (ddt, 1H, 17, 10, 7); ^{13}C δ 7.3, 28.1, 28.7, 30.4, 33.6, 33.8, 114.5, 139.0.

8-Azidooct-1-ene (**6**) Iodoalkene (**5**) (0.8653 g, 3.6 mmol, 1 eq) and sodium azide (1.1800 g, 18 mmol, 5 eq) were dissolved in DMF (18 ml). This reaction was stirred at room temperature overnight after which the solution was transferred to an Erlenmeyer flask and diluted with hexanes (75 ml). This mixture was washed with deionized water (5 X 50 ml) and the organic layer was dried with Na_2SO_4 and concentrated in vacuo to yield yellow oil. Purification by column chromatography (1.9 x 25 cm; 5% EA in Hex) furnished, after concentration of product-containing fractions, 0.5179 g (93%) of (**6**) as a clear oil. Spectral data were consistent with those previously reported.[4] R_f = 0.41 (100% Hex); ^{1}H δ 1.32-1.42 (m, 6H), 1.59 (m, 2H), 2.05 (q, 2H, 7.0) 3.26 (t, 2H, 6.9), 4.94, (bd, 1H, 10.2), 5.00 (bd, 1H, 17.1)), 5.79 (ddt, 1H, 17, 10, 7); ^{13}C δ 26.7, 28.7, 28.8, 28.9, 33.7, 51.6, 114.5, 139.0.

***S*-(8-Azidooctyl) ethanethioate** (**7**) The azidoalkene (**6**) (0.6361 g, 4.15 mmol, 1 eq), thioacetic acid (1.5353 g, 1.43 ml, 20.75 mmol, 5 eq), and AIBN (0.1363 g, 0.8 mmol, 0.2 eq) were dissolved in benzene (21 ml). The mixture was heated to reflux until complete by TLC (3.5 h).

Concentration of the cooled reaction solution gave a viscous dark oil which was purified by column chromatography (1.9 x 18 cm) using 3% EA in Hex to afford, following removal of solvent, 0.8280 g (87%) of (**7**) as a light yellow oil. Spectra are consistent with those previously reported:[5] R_f = 0.41 (100% Hex); ^{1}H δ 1.32-1.35 (m, 8H), 1.54-1.60 (m, 4H), 2.32 (s, 3H), 2.86 (t, 2H, 7.2), 3.25 (t, 2H, 6.8); ^{13}C δ 26.7, 28.8, 28.9, 29.0, 29.1, 29.2, 29.6, 30.8, 51.6, 196.1.

8-Azidooctane-1-thiol (**C8-N3**) Transesterification of the thioester was carried out by dropwise addition of concentrated HCl (3.9 mL, 47 mmol, 13 equiv) to a vigorously stirred solution of thioacetate **7** (0.8203 g, 3.6 mmol, 1 eq) in methanol (50 ml). Following completion of addition, the reaction was heated to reflux for 3 h. The reaction was then cooled to rt and diluted with deionized water (75 ml). The combined ether extracts (3 x 15 mL) were dried with Na_2SO_4 and concentrated in vacuo to give a yellow oil. Purification was carried out by column chromatography (silica, 1.9 x 23 cm) 1% EA in Hex and provided 0.6970 g (85%) of (**C8-N3**) as a light yellow oil. This molecule has been previously reported without characterization.[6] Spectra align closely with reports for a closely related mercaptoalkene.[7] R_f = 0.72 (100% Hex); ^{1}H δ 1.31-1.38 (m, 10H), 1.57-1.62 (m, 4H), 2.52 (q, 2H, 7.4), 3.25 (t, 2H, 6.9); 13C δ 24.7, 26.8, 28.3, 28.9, 29.0, 29.1, 34.0, 51.6.

Synthesis of **Tet-MB**

Tet-MB was prepared through a four-step sequence overviewed in Figure SI-4.

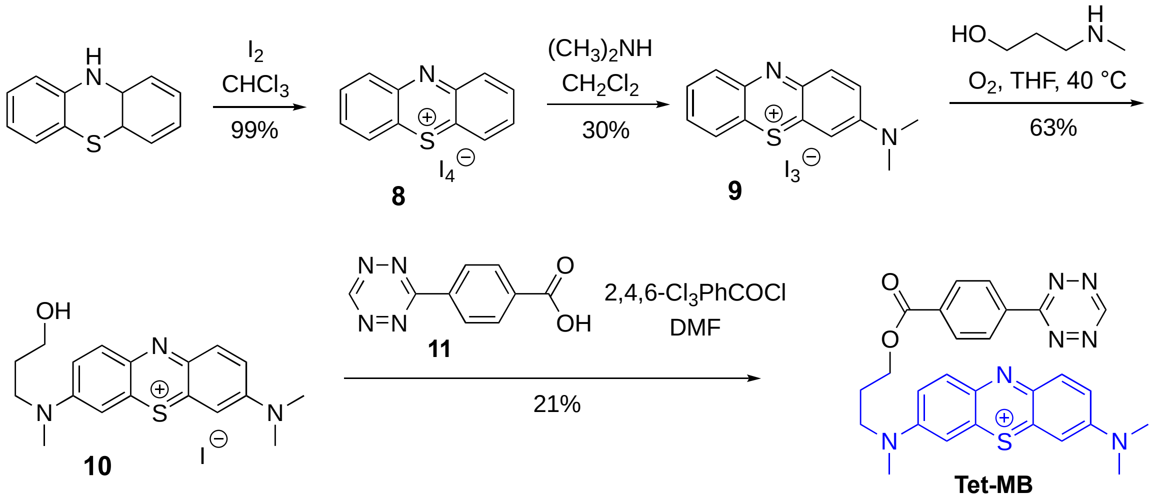

Figure SI-4. Preparation of **Tet-MB**

_**(3-(Dimethylamino)-7-((3-hydroxypropyl)(methyl)amino)phenothiazin-5-ium iodide (10)** was prepared through a two-step synthesis based upon a literature report.[8]

Phenothiazin-5-ium tetraiodide hydrate (**8**). To a solution of phenothiazine (1.165 g, 6.1 mmol, 1.0 equiv) in $CHCl_3$ (20 mL) under N_2 was dropwise added a solution of iodine (4.665 g, 18.3 mmol, 3 equiv) in $CHCl_3$ (85 mL) over a period of 1 h. After stirring overnight, the reaction was placed in an ice bath and stirred for 30 min. The cake obtained upon filtration was washed sequentially with chilled (-20° C) $CHCl_3$ (2 x 75ml) and chilled (-20° C) hexane (100 mL), at which point the pink tint of iodine was nearly absent. The black solid was collected and dried in vacuo to give **8** (4.2575 g, 99%). Spectral values for the product matched a literature report.[8] R_f = 0.68 (10% MeOH/CH_2Cl_2) ^{1}H (DMSO-d^6) δ 7.61 (t, 1H, 8), 7.72 (t, 1H 6.8), 7.93 (d, 1H, 7.6), 8.05 (m, 3H) and 8.51 (m, 2H). ESI calc. for C_{12}H8NS$^+$: (M)$^+$ calc: 198.0377; found:198.0376. MP: 176-178°C.

3-(Dimethylamino)phenothiazin-5-ium triiodide (**9**). To a solution of **8** (4.6910 g, 6.65 mmol, 1 eq.) in 65 ml of CH_2Cl_2 was added 6.65 ml (13.1 mmol, 2 eq.) of a 2M solution of dimethylamine in methanol over a period of 6 h. The reaction was then cooled in an ice bath and stirred for an additional 30 minutes. The cake obtained upon filtration was washed with chilled (-20°C) CH_2Cl_2 (2 x 75 ml). The resulting green-black solid (1.1321g, 30%) was collected and dried in vacuo to afford **9**. Collected characterization was in agreement with those found in the literature.[8, 9] R_f = 0.36 (10% MeOH/CH_2Cl_2) ^{1}H (acetone-d^6) δ 3.83 (s, 3H), 3.87 (s, 3H), 7.89 (m, 2H), 8.03 (s, 1H), 8.10 (d, 1H, 8.7), 8.15 (s, 2H) and 8.27 (d, 1H, 5.5 Hz ; ^{13}C (DMSO-d6) δ 43.44 (s), 43.90 (s), 110.32 (s), 126.73 (s, 2C), 126.90 (s, 2C), 130.45 (s), 133.85 (s), 135.16 (s, 2C), 140.47 (s, 2C), and 156.77 (s). ESI calc. for $C_{14}H_{13}N_2S^+$: (M)$^+$: 241.08; found: 241.0791; MP: 139-141°C (lit. 149-150).[9]

3-(Dimethylamino)-7-((3-hydroxypropyl)(methyl)amino)phenothiazin-5-ium iodide (**10**): A septum capped round bottom flask containing 0.235 mmol (0.1460 g, 1 eq.) of phenothiazinium triiodide **9** was evacuated and then back filled with O_2 via a needle attached to a balloon-fitted syringe barrel. The solid was then dissolved in THF (4 ml) and a solution of 3-methylamino-1-propanol (0.0440 g, 0.494 mmol, 2.1 eq.) in tetrahydrofuran (0.5 mL) was introduced via syringe. The reaction was covered in foil and stirred at 40°C (oil bath) until material had disappeared (TLC, 3 days); the product was observed as a dark blue spot which eluted in 10% methanol/CH_2Cl_2. The reaction was then concentrated in vacuo and the residue re-dissolved in 1% methanol in CH_2Cl_2. Silica (2.0 g) was added, and the suspension was concentrated in vacuo. The product-laden silica was added directly atop a previously packed and wetted (2% methanol/CH_2Cl_2) 2 x 25.4 cm column of silica and the product eluted with a gradient of 2% (600 mL) to 3% (300 mL) to 4% (300 mL) methanol/CH_2Cl_2. The dark blue solution of the product was reconcentrated to a volume of approx. 5 ml and then cooled in an ice bath. Diethyl ether was slowly added (10 ml per min.) to initiate recrystallization. After 100 ml of ether had been added, the suspension was placed in a -20°C freezer for 30 minutes and then filtered through 0.2 micropore Nylon with a 30 ml diethyl ether wash to furnish 0.2023 g (63% yield) of **10** as a dark blue solid (mp 153-156°C): R_f = 0.41 (10% MeOH/CH_2Cl_2) IR (thin film) 3436 (br), 2375, 2342, 1600, 1390, 1335 and 1140 cm^{-1}; ^{1}H (MeOD, 700 MHz) δ 1.95 (m, 2H), 3.37 (m, 3H), 3.39 (m, 6H) 3.68 (t, 2H, 1.4), 3.85 (t, 2H, 1.8), 7.35 (s, H), 7.38 (m, 1H) 7.47 (d, 1H, 2.4) and 7.51 (d, 1H, 2.0) 7.92 (d, 2H, 2.4); ^{13}C (MeOD, 175 MHz) δ 38.77, 40.14, 50.18, 58.26, 105.97, 105.99, 118.59, 118.66, 134.55, 134.57, 134.70, 135.57, 135.60, 135.63, 138.11, 138.19,

153.98, 154.42. ESI calc. for $C_{18}H_{22}N_3OS^+$: $(M)^+$: 328.4535; found: 328.1475. UV (DCM): l_{max} = 665 nm (ε = 54,000 $L{\cdot}mol^{-1}{\cdot}cm^{-1}$).

4-(1,2,4,5-Tetrazine-3-yl)benzoic acid (**11**) was prepared by an adaptation of a published procedure employing a modified workup.[10] 4-Cyanobenzoic acid (1.1770 g, 8 mmol, 1 eq.), formamidine acetate (4.1644 g, 40 mmol, 5 eq.), and zinc triflate (1.4540 g, 4 mmol, 0.5 eq.), all solids, were stirred vigorously while hydrazine hydrate (10.2560 g, 10.0 ml, 320 mmol, 40 eq.) was added dropwise. Following this addition, a rubber septum was placed on the rbf with N_2 inlet, and the resulting cloudy, white solution was stirred vigorously. After two days the solution had taken on a bright yellow, opaque color. The reaction was cooled to 0°C and a 0°C solution of sodium nitrite (5.5190 g, 80 mmol, 10 eq.) in deionized water (25 mL) was added; the resulting mixture was transferred to a 500 ml Erlenmeyer flask chilled in an eye bath. A 6M HCl, prechilled in an eye bath, was added dropwise (warning: perform in fume hood) until the reaction solution reached pH ~ 1 (indicator paper). The acidification was accompanied by a color change from yellow to bright pink and the release of nitrogen oxides. The solution was stirred for 20 mins at 0°C and then filtered and washed with deionized water (2 x 20 ml) and iPrOH (2 x 40 ml). The resulting pink cake was transferred to a 250 ml Erlenmeyer flask with 100 ml iPrOH and stirred overnight at RT. The pink solution was again filtered and wash with iPrOH until the filtrate ran clear. The pink cake was dried under high vacuum to give (0.8260 g, 51%) of (**11**) as a pink solid (decomp at 255°C; mp not previously reported): ^{1}H (THF-d8) δ 8.30 (d, 2H 7.7), 8.74 (d, 2H, 7.7) and 10.40 (s, 1H); ^{13}C (THF-d8) δ 127.75 (s, 2C), 130.33 (s, 2C), 134.71 (s), 136.18 (s), 158.40 (s), 165.92 (s) and 166.08 (s). UV (solvent): MeCN λ_{max} = 296 nm (ε = 9600 L·mol−1·cm−1).

3-((3-((4-(1,2,4,5-Tetrazin-3-yl)benzoyl)oxy)propyl)(methyl)amino)-7-(dimethylamino)-phenothiazin-5-ium iodide (**Tet-MB**). In a 30 ml vial with septum cap and under nitrogen, tetrazine benzoic acid **11** (0.0290 g, 0.14 mmol, 1 eq.) was dissolved in a mixture of 0.8 ml DMF and triethylamine (0.0280g, 0.28 mmol, 2 eq.). 2,4,6-Trichlorobenzoyl chloride (33 µl, 0.051 g, 0.21 mmol, 1.5 eq.) was added and the mixture was stirred for 2 h. 4-Dimethylaminopyridine (DMAP, 0.0188 g, 0.154 mmol, 1.1 equiv) was added followed by phenothiazonium iodide **10** (0.0699 g, 0.154 mmol, 1.1 equiv) in 0.3 ml additional DMF and the reaction was stirred for 18 hrs. TLC revealed a dark blue spot which eluted in 10% methanol/CH_2Cl_2 with a higher R_f than **10**. The reaction was then diluted with 20 ml CH_2Cl_2, and the resulting solution directly loaded onto a 2 x 25.4 cm silica column. Elution using a gradient of 0% (100 mL) to 2% (200 mL) to 4% (600 mL) of methanol/CH_2Cl_2 resulted in the emergence of violet-colored fractions which were concentrated to a residual volume of approximately 5 mL. The solution was cooled in an ice bath and slowly diluted with ether (10 ml per min.) to initiate recrystallization. After 100 ml of ether had been added, the suspension was placed in a -20°C freezer for 30 minutes and then filtered through a Nylon filter (200 micron). The solid was washed with ether (30 ml) to furnish 0.0182 g (21% yield) of **10** as a purple solid (decomp at 186°C): R_f = 0.53 (10%MeOH/CH_2Cl_2) ^{1}H (CD_2Cl_2, 700 MHz) δ 2.34 (m, 2H), 3.44 (s, 9H), 3.99 (t, 3H, 7.04), 4.55 (t, 3H, 5.76), 7.34 (m, 4H), 7.94 (d, 2H, 9.44), 8.26 (d, 2H, 8.08), 8.71 (d, 2H, 8) and 10.34 (s, 1H); ^{13}C (CD_2Cl_2,

175 MHz) δ 13.87 (s), 26.66 (s), 29.67 (s), 40.22 (s), 41.70 (s), 50.78 (s), 62.68 (s), 106.34 (s), 106.41 (s), 118.36 (s), 118.75 (s), 128.23 (s), 130.27 (s), 134.75 (s), 134.93 (s), 135.86 (s), 138.68 (s), 138.73 (s), 153.64 (s), 154.64 (s), 154.21 (s), 158.13 (s), 165.39 (s) and 165.91 (s). ESI calc. for $C_{27}H_{26}N_7O_2S^+$: $(M-I)^+$: 512.1863; found: 512.1858. UV (DCM): λ_{max} = 655 nm

Synthesis of **Tet-Fc**

Tet-Fc was prepared as the acetate salt by the three-step sequence overviewed in Figure SI-5.

Figure SI-5. Preparation of Tet-Fc

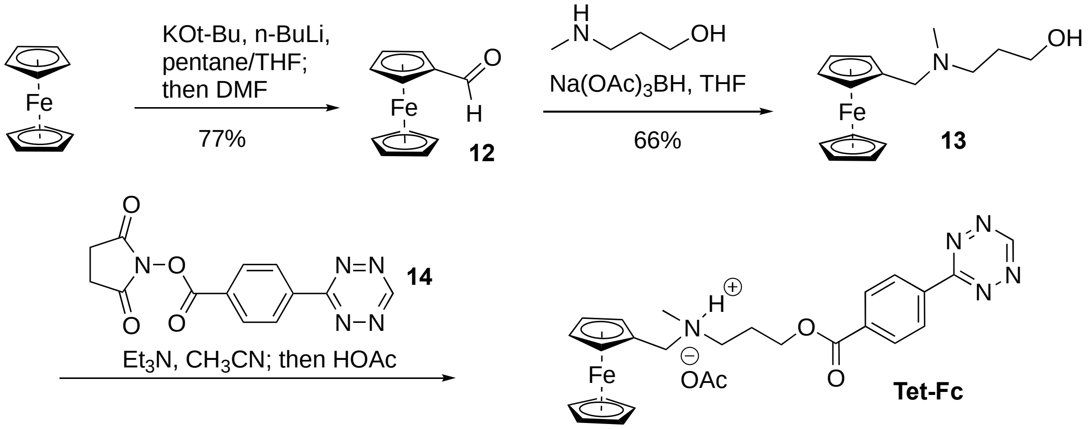

Ferrocene-2-carboxaldehyde (**12**) was prepared by a modification of a reported procedure.[11] A solution of *tert*-butyllithium in pentane (nominally 1.7M, 5.90 ml, 10 mmol, 2 eq.) was added dropwise over ten minutes into a flame dried flask containing a clear orange – 78°C solution of ferrocene (0.9340 g, 5.0 mmol, 1 eq; recrystallized from boiling hexanes) and potassium tert-butoxide (0.0733 g, 0.65 mmol, 0.13 eq.) in THF (50 ml). The resulting opaque orange mixture was stirred for one hour at which point DMF (0.89 ml, 11.5 mmol, 2.3 eq.) was added. The cooling bath was removed, and the temperature allowed to rise to -40°C over a period of 20 minutes. The solution became translucent and retained its orange color until quenched by addition of DI water (75 ml), at which point the solution became dark red. The combined organic layers derived from extraction with CH_2Cl_2 (4 x 25 ml) were washed with brine (1 x 10ml) and then dried with Na_2SO_4. Concentration in vacuo produced a thick red oil which was purified by column chromatography (Silica, 1.9 x 25 cm), using CH_2Cl_2 to elute any unreacted ferrocene followed by 5% MeOH in CH_2Cl_2 to elute the desired product (**12**) as a dark red oil (0.8217 g, 77%) which solidified upon storage at -20°C: mp 117-119°C (literature, 118-120); R_f = 0.92 (10% MeOH:CH_2Cl_2); IR (thin film) 3094 (w), 2823 (w), 2756 (w) 1676 (s), 1659 (s) cm^{-1}; ^1H δ 4.27 (s, 5H), 4.61 (s, 2H), 4.80 (s, 2H), 9.96 (s, 1H); ^{13}C δ 69.8, 73.3, 77.3, 79.5, 193.6.

3-(*N*-(2-Ferrocenylmethyl)-N'-methyl)aminopropan-1-ol (**13**): Ferrocenecarboxaldehyde (**12**, 0.6431 g, 3.00 mmol, 1 eq.) and $MgSO_4$ (0.5422 g, 4.5 mmol, 1.5 eq) were dissolved with stirring in THF (15 ml, 0.2M) within in a flame-dried flask which had been back-filled with nitrogen. Once the aldehyde was in solution, *N*-methyl-3-aminopropanol (0.32 ml, 3.3 mmol, 1.1 eq.) and sodium triacetoxyborohydride (1.5261 g, 7.2 mmol, 2.4 eq.) were sequentially added. The reaction was stirred while protected from light (aluminum foil) for 20 h, at which point starting material could no longer be detected (TLC). The reaction was quenched with 1M NaOH (30 ml). The resulting mixture was stirred for 30 min and then diluted with sat. aq. NH_4Cl (40 ml). The combined CH_2Cl_2 extracts (3 x 30 ml) were washed with brine (1 x 10ml) and dried over Na_2SO_4. T/he residue obtained after concentration in vacuo was purified by silica column chromatography (1.9 x 25 cm, 5-20% MeOH in CH_2Cl_2) to furnish 0.5761 g (66%) of alcohol **13** as a yellow-brown oil which solidified upon storage at 20°C. R_f = 0.47 (10% MeOH:CH_2Cl_2); IR (thin film) 3092 (w), 2943 (m), 2845 (m), 2795 (m) 1463 (m) cm^{-1}; ^{1}H δ 1.68 (p, 2H, 5.4), 2.19 (s, 2H), 2.58 (t, 2H, 5.6), 3.44 (s, 2H), 3.75 (t, 2H, 5.2), 4.12 (s, 5H), 4.12 (d, 2H, 1.8), 4.17 (t, 2H, 1.8); ^{13}C δ 27.7, 41.6, 57.1, 57.3, 64.7, 68.3, 68.7, 70.3, 80.7. HRMS-EI calc. for $C_{15}H_{21}FeNO$ (M)+: 287.0973; found: 287.0967.

3-(*N*-(2-Ferrocenylmethyl)-N'-methyl)aminopropan-1-yl, 4-(1,2,4,5-tetrazin-3-yl)benzoate (**Tet-FC**) could be prepared using either of two equally useful procedures:

One step procedure (Yamaguchi Esterification): A 25 ml rbf equipped with stir bar was flame dried, evacuated, and back filled with nitrogen. 4-(1,2,4,5-Tetrazin-3-yl)benzoic acid (0.0400 g, 0.2 mmol, 1 eq.), triethylamine (0.0405 g, 0.4 mmol, 2 eq.), and DMF (3 ml) were added sequentially, and the resulting solution was allowed to stir for 10 minutes or until the acid had fully dissolved. 2,4,6-Trichlorobenzoyl chloride (0.0731 g, 47 µl, 0.3 mmol, 1 eq.) was added and the reaction allowed to proceed at room temperature for 0.5 h. 3-(*N*-(2-Ferrocenylmethyl)-N'-methyl)aminopropan-1-ol (**13**) (0.1723 g, 0.6 mmol, 3 eq.) and *N,N'*-dimethylaminopyridine (0.0510 g, 0.42 mmol, 2.1 eq.). were added and the reaction was covered in aluminum foil and stirred. After 24 h, the crude reaction was concentrated and submitted to a tedious column chromatography (1.9 x 25.4 cm) using a gradient of 0-5% MeOH in CH_2Cl_2 to furnish, following concentration in vacuo, 0.0474 g (50%) of ester **Tet-FC** as a pinkish-red film which clung to the walls of the flask: R_f = 0.56 (10% MeOH:CH_2Cl_2); IR (thin film) 3282 (br, s), 1716 (m) cm^{-1}; ^{1}H δ 2.05 (p, 2H, 6.8) 2.32 (s, 3H), 2.60 (apparent s, 2H), 3.59 (s, 2H) 4.12 (s, 5H), 4.13 (s, 2H), 4.21 (s, 2H), 4.40 (t, 2H, 6.3), 8.21 (d, 2H, 8.3), 8.70 (d, 2H, 8.3),10.28 (s, 1H); ^{13}C δ 26.1, 41.3, 52.3, 53.4, 56.9, 63.5, 68.6, 68.7, 70.4, 128.2, 130.5, 134.2, 135.5, 158.0, 165.7, 166.0. HRMS-EI calc. for $C_{24}H_{25}FeN_5O_2$ (M)+: 471.1358; found: 471.1359. UV (MeCN): λ_{max} = 263 nm (ε = 23,000 $L\cdot mol^{-1}\cdot cm^{-1}$)

Two step procedure:

2,5-Dioxopyrrolidin-1-yl 4-(1,2,4,5-tetrazin-3-yl)benzoate (**14**). 4-(1,2,4,5-Tetrazin-3-yl)benzoic acid (0.1011 g, 0.5 mmol, 1 eq.), was placed in a reaction flask which had been flame-dried and then cooled under N2. THF (48 ml) was added, and the stirred reaction was heated to ~ 50°C until the acid dissolved. *N*-Hydroxysuccinimide (0.1726 g, 1.5 mmol, 3 eq.),

N,N'-dimethylaminopyridine (0.0061 g, 0.05 mmol, 0.1 eq.), and dicyclohexylcarbodiimide ds(0.1135 g, 0.55 mmol, 1.1. eq.) were added sequentially and the reaction was stirred overnight. The completed reaction was concentrated and subjected to column chromatography (3.8 x 35 cm) with a gradient of 0-2% MeOH in CH_2Cl_2. Collected fractions were concentrated in vacuo to provide 0.0698 g (46%) of a bright pink solid which was contaminated with 1,3-dicyclohexylurea. The inseparable mixture was directly used in the proceeding reaction. R_f = 0.92 (10% MeOH:CH_2Cl_2); IR (thin film) 2919 (m), 1769 (s), 1731 (s) cm-1; ^{1}H δ 2.95 (s, 4H) 8.38 (d, 2H, 8.6), 8.80 (d, 2H, 8.6),10.32 (s, 1H); ^{13}C δ 25.8, 29.4, 128.7, 129.2, 131.5, 137.4, 158.2, 161.3, 169.1. HRMS-EI calc. for $C_{13}H_9N_5O_4$ $(M)^+$: 299.0655; found: 299.0648. MP: sublimes at 186-190°C.

3-(N-(2-ferrocenylmethyl)-N'-methyl)aminopropan-1-yl, 4-(1,2,4,5-tetrazin-3-yl)benzoate, acetic acid salt (**Tet-FC**)

The *N*-hydroxysuccinimide ester (2,5-dioxopyrrolidin-1-yl 4-(1,2,4,5-tetrazin-3-yl)benzoate, **14**) (0.0450 g, 0.16 mmol, 1 eq.) was dissolved in ACN (6 mL) within a 25 ml flame-dried RBF equipped with a stir bar. Alcohol **13** (3-(*N*-(2-ferrocenylmethyl)-N'-methyl)aminopropan-1-ol, 0.0919 g, 0.32 mmol, 2 eq.) and triethylamine (0.0640 g, 80 µl, 0.64 mmol, 4 eq) were added and the reaction, protected from light (foil) was stirred at room temperature for 18 hours. The completed reaction was concentrated and purified through relatively facile column chromatography (1.9 x 12.7 cm) 0-4% MeOH in CH_2Cl_2, with fractions collected in test tubes pretreated with 3-5 drops of glacial acetic acid. The concentration of the collected fractions provided 0.051 g (68%) of ester **Tet-FC** as a pinkish-red film lining the flask. R_f = 0.56 (10% MeOH:CH_2Cl_2); IR (thin film) 3282 (br, s), 1716 (m) cm^{-1}; ^{1}H δ 2.05 (p, 2H, 6.8) 2.32 (s, 3H), 2.60 (apparent s, 2H), 3.59 (s, 2H) 4.12 (s, 5H), 4.13 (s, 2H), 4.21 (s, 2H), 4.40 (t, 2H, 6.3), 8.21 (d, 2H, 8.3), 8.70 (d, 2H, 8.3),10.28 (s, 1H); ^{13}C δ 26.1, 41.3, 52.3, 53.4, 56.9, 63.5, 68.6, 68.7, 70.4, 128.2, 130.5, [12]134.2, 135.5, 158.0, 165.7, 166.0. HRMS-EI calc. for C24H25FeN5O2 (M)+: 471.1358; found: 471.1359. UV (MeCN): l_{max} = 263 nm (e = 23,000 L·mol^{-}1·cm^{-}1).

[1] Cañete, S. J. P.; Yang, W. and Lai, R. Y. Folding-based electrochemical DNA sensor fabricated by "click" chemistry. *Chemical Commun.* **2009**, 4835-4837.

[2] Cañete, S. J. P. and Lai, R. Y. Fabrication of an Electrochemical DNA Sensor Array via Potential-Assisted "Click" Chemistry. *Chem. Commun.*, **2010**, *46* (22), 3941−3943.

[3] a. Sih, C. J.; Salomon, R. G.; Price, P.; Sood, R.; Peruzzotti, G. Total synthesis of prostaglandins. VI. Stereospecific total synthesis of prostaglandins via reaction of .alpha.-alkylcyclopentenones with organocuprates. *J. Am. Chem. Soc.* **1975**, 97, 857–865; b. Negishi, E.I.; Rousset, C.J.; Choueiry, D.; Maye, J.P.; Suzuki, N. and Takahashi, T. Zirconium-catalyzed and zirconium-promoted cyclization reactions of non-conjugated dienes with alkylmagnesium halides to give cycloalkylmethylmagnesium derivatives 1998. *Inorg. Chim. Acta*, 280 (1−2), 8−20.

[4] a. Steinmann, M.; Wagner, M.; Wurm, F. R. Poly(phosphorodiamidate)s by Olefin Metathesis Polymerization with Precise Degradation. *Chem. Eur. J.* **2016**, *22*,17329–17338; b. Ourri, B.; Tillement, O.; Tu, T.; Jeanneau, E.; Darbost, U.; Bonnamour, I. Copper complexes bearing an NHC–calixarene unit: synthesis and application in click chemistry. *New J. Chem.* **2016**, *40*, 9477−9485.

[5] a. Kenfack, G. T.; Schinkovitz, A.; Babu, S.; Elouarzaki, K.; Dias, M.; Derbré, S.; Helesbeux, J. J.; Levillain, E.; Richomme, P.; Séraphin, D. Triazolobithiophene Light Absorbing Self-Assembled Monolayers: Synthesis and Mass Spectrometry Applications. *Molecules* **2011**, 16, 8758–8774; b. Molloy, J. K.; Licheneau, C.; Karimdjy, M. M.; Agnese, F.; Mattera, L.; Gateau, C.; Reiss, P.; Imbert, D.; Mazzanti, M. Sensitisation of visible and NIR lanthanide emission by InPZnS quantum dots in bi-luminescent hybrids. *Chem. Commun.*, **2016**, 52, 4577−4580.

[6] Han, X.; Bian, S.; Liang, Y.; Houk, K. N.; Braunschweig, A. B. Reactions in Elastomeric Nanoreactors Reveal the Role of Force on the Kinetics of the Huisgen Reaction on Surfaces. *J. Am. Chem. Soc*. **2014**, *136*, 10553−10556.

[7] Lin, Y-Y.; Tsai, S-C.; Yu, S. J. Highly Efficient and Recyclable Au Nanoparticle-Supported Palladium(II) Interphase Catalysts and Microwave-Assisted Alkyne Cyclotrimerization Reactions in Ionic Liquids. *J. Org. Chem.* **2008**, *73*, 4920−4928.

[8] New, O. M.; Dolphin, D. Design and Synthesis of Novel Phenothiazinium Photosensitiser Derivatives. *Eur. J. Org. Chem*. **2009**, 2675−2686.

[9] Strekowski, L.; Hou, D.-F.; Wydra, R.L.; Schinazi, R.F. A synthetic route to 3-(dialkylamino)phenothiazin-5-ium salts and 3,7-disubstituted derivatives containing two different amino groups. *J. Het. Chem.* **1993**, 30 (60), 1693−1695.

[10] Karver, M. R.; Weissleder, R.; Hilderbrand, S. A. Synthesis and Evaluation of a Series of 1,2,4,5-Tetrazines for Bioorthogonal Conjugation. *Bioconj. Chem*. **2011**, 22, 2263−2270.

[11] Sanders, R.; Mueller-Westerhoff, U.T. The lithiation of ferrocene and ruthenocene: a retraction and an improvement. *J. Organometal. Chem*, **1996,** 512(1-2), 219−224.
